# Building Biocompatible Hydrogels for Tissue Engineering of the Brain and Spinal Cord

**DOI:** 10.3390/jfb3040839

**Published:** 2012-11-15

**Authors:** Emily R. Aurand, Jennifer Wagner, Craig Lanning, Kimberly B. Bjugstad

**Affiliations:** 1 Neuroscience Program and Department of Pediatrics, University of Colorado-Denver, Anschutz Medical Campus, Mail Stop 8313, 12800 E. 19th Avenue, Aurora, CO 80045, USA; Email: emily.aurand@ucdenver.edu; 2 Department of Bioengineering, University of Colorado-Denver, Anschutz Medical Campus, Mail Stop 8607, 12700 E. 19th Avenue, Aurora, CO 80045, USA; Email: jennifer.wagner@ucdenver.edu (J.W.); craig.lanning@ucdenver.edu (C.L.)

**Keywords:** brain, spinal cord, microglia, astrocytes, biocompatibility, hydrogel, tissue engineering

## Abstract

Tissue engineering strategies employing biomaterials have made great progress in the last few decades. However, the tissues of the brain and spinal cord pose unique challenges due to a separate immune system and their nature as soft tissue. Because of this, neural tissue engineering for the brain and spinal cord may require re-establishing biocompatibility and functionality of biomaterials that have previously been successful for tissue engineering in the body. The goal of this review is to briefly describe the distinctive properties of the central nervous system, specifically the neuroimmune response, and to describe the factors which contribute to building polymer hydrogels compatible with this tissue. These factors include polymer chemistry, polymerization and degradation, and the physical and mechanical properties of the hydrogel. By understanding the necessities in making hydrogels biocompatible with tissue of the brain and spinal cord, tissue engineers can then functionalize these materials for repairing and replacing tissue in the central nervous system.

## 1. Introduction

The central nervous system (CNS) is comprised of the brain and spinal cord. In attempting to re-engineer neural tissues of the CNS, two specific characteristics must be taken into account which make this system unique compared to tissues found in the rest of the body. First, the CNS is considered an “immune-privileged” tissue. The CNS immune responses are mostly governed by resident cells of the brain rather than the immune cells of the body. This means that when determining the biocompatibility of polymers, implanted cells, or devices for use in the brain, the neuroimmune response must be determined—not necessarily the peripheral immune response alone. For example, poly(methylidene malonate 2.1.2) microspheres were found to be biocompatible when injected intravenously but when implanted into the brain, the microspheres initiated a massive immune response resulting in the death of some the test subjects [1,2]. Under certain conditions, peripheral immune cells *do* pass from the periphery into the brain, such as during occasions of brain damage or in some diseases which can weaken the blood-brain barrier. In these instances, however they are not being recruited to take on primary immune responses but typically act to antagonize the neuroimmune response, such as in HIV infection or multiple sclerosis [3,4]. Thus, it is the immune response of the host brain cells that must require our initial attention when attempting neural tissue engineering. Once we understand how the CNS, under normal conditions, will respond to our attempts, we can customize our approach to accommodate disease specific nuances in immune responses. 

The second characteristic making the CNS unique is that it has a limited capacity to repair damage and grow new cells. Unlike other tissues in the body, the adult brain does not undergo widespread cell replacement, thus to be most effective, re-engineering damaged brain tissue would most likely involve implanting replacement neural cells. Unfortunately, the act of implanting these cells engages the neuroimmune system to respond to the damage created by penetrating the brain. The goal of the neuroimmune response, similar to the immune response in the body, is to mitigate the damaging elements inducing neuroinflammation, to contain the damage by building a glial scar, and finally, to activate the healing process to repair damaged, but still surviving, neural cells. In the end, most replacement cells, along with neighboring host cells, are lost during the acute neuroinflammatory response [5,6,7,8,9,10,11]. Depending on the source of the replacement cells and where they are implanted, cell survival can range from less than 1%, to only as high as 10% [6,7,8,10,12,13,14,15]. The ability to improve survival and promote replacement neural cell integration into the CNS would greatly advance neural tissue engineering.

Decades of neural cell replacement strategies used for treating the neurodegenerative disorder Parkinson’s disease revealed that neurons survive better in the host when they are implanted as a piece of donor neural tissue, rather than as dissociated cell suspensions [16,17,18,19]. This demonstrates the impact of retaining a three-dimensional (3D) environment in which cell attachments are maintained. As well, donor neural tissue pieces preserve components of the brain extracellular matrix (ECM), which can provide chemical and physical support for the implanted cells. Thus, as in other tissue engineering strategies, the ability to mimic the 3D ECM environment becomes a key element to successful tissue engineering. Unfortunately, political and ethical complications of using whole neural tissue have limited its use, and as such, current tissue engineering strategies focus on using neural stem/progenitor cell lines as a source for replacement neural cells. The use of cell lines, grown as single cell suspensions, makes the need for an artificial ECM more important.

Recently, advancements in polymer hydrogels have established new possibilities for neural tissue engineering by providing a way to recreate the ECM. Replacement neural cells encapsulated within a hydrogel may be protected from the acute neural inflammatory response, and thus, much more likely to survive [5,20,21,22,23,24,25]. Because hydrogels can be formulated from many different types of polymers and with a broad variety of material properties, they are an ideal material for replicating the 3D ECM of neural tissue [26,27,28,29,30,31,32,33,34,35,36,37].

Before hydrogels can be used to overcome the obstacles of replacement neural cell implantation, they must be appropriately designed to be biocompatible with the encapsulated neural cells and with the CNS environment. If the hydrogel is not biocompatible, the neuroimmune response to the implant may create additional damage to which the brain is not equipped to repair. This review will briefly address the issues of CNS biocompatibility and identify key considerations which should be made in building biocompatible hydrogels for neural tissue engineering.

## 2. CNS Environment and Neuroimmune Response

The CNS is composed of two main neural cell types: neurons and glia. The limited capacity for the brain and spinal cord to self-repair lies in that mature neurons are post-mitotic and thus, cannot give rise to new cells. Glial cells, however, do proliferate but can also be an obstacle in neural tissue engineering. There are three main types of glial cells: astrocytes, microglia, and oligodendrocytes. Under normal conditions, astrocytes perform a wide variety of supportive functions in the CNS. Through their interaction with the vascular system, astrocytes form a restrictive barrier through which they exchange nutrients and wastes with the rest of the body [38,39]. This astrocytic barrier, the blood-brain barrier (BBB), prevents the blood and immune cells in the body from entering the CNS. Thus, the brain and spinal cord must attend to immunogenic stimuli using resident cells, rather than relying on the immune system of the body. Microglia cells are the resident neuroimmune cells in the CNS. During fetal development, microglia differentiate from the same cell lineage as macrophages. Both cell types have similar immune functions, but reside in two distinct areas [40,41,42]. Oligodendrocytes in the CNS produce the myelin sheath which wraps neuronal axons to moderate signal transduction [38,43,44]. Oligodendrocyte impairment is the basis of neurodegenerative disorders such as multiple sclerosis and the leukodystrophies [45,46,47,48]. In addition to the neurons and glia, a very small population of neural stem cells can also be found in the subventricular zone of the lateral ventricles and the subgranular zone of the dentate gyrus in adult brains [49,50,51,52,53]. These neural stem cells produce neurons and glia that replace cells of the olfactory bulb and the hippocampus where neurogenesis is necessary for continued function [49,50,51,52,53]. However, this production of mature neural cells is very limited and cannot compensate for the amount of damage typically seen in most CNS injuries and disorders, thus the need to improve neural cell replacement strategies [50,51,52]. 

Just as with the body, an immune response is triggered when damage occurs to neural tissue. This can arise from the compressive damage of traumatic brain injury (TBI), prolonged cell death due to disease pathology as in Parkinson’s disease, or simply the act of inserting a needle to implant a medical device or replacement cells. For this review, we are most concerned about triggering the neuroimmune response through the implantation of biomaterials. During implantation, the blood-brain barrier is breached and there is an influx of plasma, water (*i.e.*, edema), and peripheral blood-borne cells into the cranial compartment [39,54]. This influx of fluid and of foreign blood cells including macrophages and lymphocytes can initiate inflammation and trigger the neuroimmune response [54,55]. In the brain, a neuroimmune response is signified by the recruitment of microglia and astrocytes [40,41,42]. The reactivity of both cell types is most noticeable by a change in cell morphology. Un-reactive glia have small cell bodies with long, thin processes. Reactive glia have enlarged cell bodies, with a shortening and thickening of their processes [40,56,57]. Upon becoming reactive, microglia become phagocytic and release reactive toxins in an attempt to eliminate foreign entities [40,41,58]. Astrocytes engage in absorbing excess fluid and excitotoxic chemicals. This acute response is elevated up to seven days after the initial insult and it is at this time that most implanted replacement cells become collateral damage [6,55,58,59,60]. The role of peripheral immune cells, such as peripheral macrophages and lymphocytes, in this neuroimmune process is difficult to identify but it is generally assumed that these cells are eliminated early in the neuroimmune response as foreign cells. However, Yang *et al.* [61] found an infiltration of T-cells residing up to 8 weeks within the implanted hydrogels but no peripheral immune cells were found in the surrounding tissue. Eventually the immune response shifts to repair processes [39,42,56]. Both microglia and astrocytes begin to produce growth factors and anti-inflammatory cytokines to repair damage to the brain or spinal cord [38,39,40,41,42,56,58,62,63].

Extracellular matrix proteins secreted by cells at the wound site develop into a glial scar. In the spinal cord, the scar prevents axons from reconnecting across the site of damage, limiting the recovery of motor function. In the brain, the effects of glial scarring are less well understood. Haberler *et al.* [64] analyzed brains from eight Parkinson’s disease patients implanted with electrodes for deep brain stimulation. In each case, the brain tissue surrounding the electrode did not appear to have any long term glial activation but a glial scar encased the electrode leaving a well defined cavity in the brain. Chondroitin-sulfated proteoglycans (CSPG), laminin, collagen, and fibronectin can all be found in the glial scar. CSPGs are inhibitory to the outgrowth of neuronal axons [65,66,67,68,69,70,71,72]. Of note, is the presence of laminin, collagen, and fibronectin in the glial scar. These three components are abundant in the ECM of bodily tissues however they are generally not found in undamaged CNS tissue because of their overly fibrous nature. However, *in vitro* studies have shown that they promote axonal outgrowth from neurons plated on laminin, collagen, and/or fibronectin coated plates [65,66,68,69,70,71,72]. The role of these fibrous components on axonal outgrowth, nerve repair, and glial scarring has yet to be firmly established. Studies by Davies *et al.* [62,73] suggest that the type of astrocyte involved in forming the glial scar is the primary determinant of growth permissiveness. Some astrocytes produce less CSPGs when activated and tend to promote fibrous alignment within the scar tissue, which seems to be more conducive to axonal growth across the scar [73].

In addition to a overall glial scarring, the number and degree of reactivity of the surrounding astrocytes and microglia involved in the immune response can be considered an indicator of the level of immunorejection [60] and can therefore be used as a tool to determine *in vivo* biocompatibility [55,74,75]. For example, rat brains implanted with a more quickly degrading poly(ethylene glycol) (PEG)-based hydrogel had fewer microglia but more astrocytes in the surrounding brain, than brains implanted with a more slowly degrading hydrogel of the same composition. However, most of the astrocytes and microglia had a reactive morphology surrounding the more quickly degrading hydrogel suggesting that the neuroimmune response was still activated [55]. Yang *et al.* [61], also demonstrated the *in vivo* reactivity of astrocytes and microglia in response to a biocompatible hydrogel. Levels of astrocyte reactivity around the implanted hydrogel were shown to be comparable to those around a saline injection. As well, microglia were shown to be nearly absent around the implanted hydrogel after eight weeks, similar to the saline injection [61]. In traumatic brain injury, the microglial presence can remain elevated long after the initial injury [76,77,78,79]. Thus, to determine the biocompatibility of a biomaterial with brain or spinal cord tissue, the acute, as well as the long-term behavior of the glial cells should be determined for as long as the desired biomaterial will be *in situ*.

## 3. Chemistry and Polymer Components

There are many considerations which need to be made with regards to hydrogel chemistry. It is important to recognize that the monomers or polymers chosen will not only impact the material properties (physical and mechanical), but will also affect the biocompatibility of the hydrogel. A wide variety of synthetic polymers have been shown to be biocompatible in the body, such as polyesters and acrylates [60,80,81,82,83,84]. Natural polymers, such as poly (amino acids) and hyaluronic acid, have also been modified to form biocompatible hydrogels [83,85,86,87,88,89]. It is necessary that the polymers used are biocompatible in their precursor state, as a fully polymerized hydrogel, and in any form that may occur with degradation. A number of materials have been investigated which have demonstrated adverse reactions with cells *in vitro* or with tissue *in vivo* [1,60,90,91], and thus should be avoided when choosing materials. For example, the degradation products of poly(methylidene malonate 2.1.2) microspheres were found to induce pronounced neuroimmune responses and even animal death [1]. Initially, poly (methylidene malonate 2.1.2) microspheres implanted into rat brain did not induce a neuroimmune response, suggesting that the polymerized hydrogel was biocompatible. However, when the microspheres began to degrade several months later, there was large scale cell loss and reactive glial response, ultimately causing tissue lesions and death [1].

Polymerization of a hydrogel is dependent on the chemistry of the precursors, which in and of themselves, must be biocompatible. However, the methods of polymerization, dictated by the chemistry, can also affect biocompatibility. Photopolymerization, which often uses a photoreactive catalyst for bond formation between polymers, can lead to the formation of free-radicals which can be damaging to encapsulated cells [92,93,94,95]. Dopaminergic neurons are especially susceptible to free-radicals, as they are already under higher levels of oxidative stress resulting from dopamine synthesis [96]. Thus, when treating Parkinson’s disease, it may be prudent to use polymers where the polymerization process produces little or no free-radicals. Polymerization processes which require pH or temperature changes can be highly beneficial if the pH or temperature at which polymerization occurs matches physiological conditions. Hydrogels which are liquid at non-physiological conditions, but then polymerize *in situ* at physiological conditions may prove to be very helpful because they can be injected as liquids, minimizing the invasiveness of the implantation procedure [27,89,97,98,99,100,101]. Conova *et al.* [102] found that thermosensitive hydrogels based on poly (*N*-isopropylacrylamide) could be injected into the damaged spinal cord as a viscous liquid which polymerized *in situ*. These hydrogels did not antagonize the neuroimmune response and supported encapsulated cell survival and the controlled release of growth factors [102].

Just as biocompatibility is dependent on polymerization precursors and the method in which polymerization occurs, degradation also contributes to biocompatibility through hydrogel degradation products and the method of degradation. Many biodegradable polymers are broken down into constituent elements through a combination of enzymatic processes and/or hydrolysis; these elements are then bioeliminated (if water soluble), or cleared by cellular metabolism [27,94,103,104,105,106,107,108,109]. As stated previously, poly(methylidene malonate 2.1.2) microspheres induced a strong neuroimmune response only during the degradation process into its constituent elements, which have an acidic nature [1]. However, studies done in the past suggested that poly (methylidene malonate 2.1.2) microspheres were biocompatible in the body when injected intravenously or when used to culture fibroblast cells [2]. These studies nicely illustrate not only the importance of the degradation products, but also the unique response of the brain. Biocompatibility in the body is not the same as biocompatibility in the CNS.

The host tissue into which a hydrogel is implanted, whether it is body tissue or the CNS, impacts the degradation process; specifically, the access of water, the type of immune cells present, and the enzymes the tissue produces [103,105,107,109,110]. The brain is about 80% water, with higher water content in grey matter than in white matter [111,112]. Conditions of stroke, tumor, hydrocephalus, or edema can increase the amount of water in the brain [111,112]. By comparison, heart tissue is also about 80% water, whereas lung tissue has a very low (6%–7%) water content [113,114]. Higher water content in a tissue will typically result in faster degradation due to hydrolysis or because of the increased fluid flow which replenishes degrading enzymes [105,115]. As described, in the brain, the immune response is primarily governed by microglia and astrocytes. In response to a lactic acid and PEG-based hydrogel implanted into the brain, microglia were found to have infiltrated the hydrogel, facilitating the enzymatic degradation process, whereas astrocytes only extended their processes into the hydrogel [55]. Furthermore, hydrogels composed of natural polymers similar to those found in the host tissue—such as hyaluronic acid (HA), which forms the backbone of the ECM in the brain—will be more likely to undergo enzymatic degradation via endogenously produced enzymes [116,117]. In the case of HA, the major degrading enzyme is hyaluronidase, which is secreted by neurons and some glia [118].

The ability for a hydrogel to degrade is necessary in the brain because glial scarring can occur around permanent implants [119,120,121,122]. Scarring inhibits the repair and reconnection of neural circuitry, which is especially apparent in spinal cord injury [123,124,125]. The ability to degrade is dependent on precursor chemistry. The molecular weight of the individual hydrogel components impacts the degradation rate: typically, higher molecular weight polymers produce denser hydrogels, thus slowing degradation. In a similar fashion, hydrogels with greater total percent polymer content degrade more slowly [28,103,105,106,115,126,127,128]. Simply, the rate at which a hydrogel degrades can alter the neuroimmune response. In rat brain tissue, it was found that faster degrading hydrogels invoked a neuroimmune response similar in degree and rate of resolution as observed in brains given a needle penetration only [55]. By comparison, slowly degrading hydrogels invoked less of an acute response, but during the chronic phase, maintained a larger non-reactive glial population [55]. This effect is also demonstrated by Tysseling, *et al.* [129], using self-assembling peptide amphiphile nanofibers with the laminin epitope IKVAV. The authors demonstrate the regeneration of spinal cord axonal fibers across an injury site following the degradation of the nanofibers. While nanofiber degradation was necessary for axonal outgrowth, the authors suggest that the nanofibers also may have primed the lesion site for this regeneration [129].

To improve biocompatibility, the hydrogel chemistry can be altered with the addition of certain molecules or monomers [27,81,130,131,132,133,134,135,136,137]. The most commonly referenced additions are heparin or gelatin, which mimic many ECM proteoglycans, and the amino acid sequence Arg-Gly-Asp (RGD) [5,87,130,131,138,139,140,141]. The addition of heparin, gelatin, or RGD sequences can facilitate cell attachment to the hydrogel, increasing the ability for neural cells to extend processes, migrate, and differentiate [132,135,142,143,144,145]. Growth or trophic factors can be added to a hydrogel to further encourage proliferation, migration, neurite extension, and differentiation [22,146,147,148,149,150,151,152,153,154]. Other chemical groups may be added to counter-act a property which negatively impacts biocompatibility. For example, the addition of lactic acid to a photopolymerizing PEG hydrogel may help to neutralize free-radicals and improve neural cell survival [92,95]. The additions can be permanently incorporated by covalent attachment to the hydrogel polymer backbone, or freely incorporated into the hydrogel and released by diffusion and/or degradation [27,134,135,137].

It is important that hydrogel components and additions, such as the presence or absence of replacement cells, are applicable to CNS tissue. A common biomaterial based on peripheral ECM components, collagen, laminin, and fibronectin, is Matrigel, derived from a murine sarcoma cell line. It is successfully used for *in vitro* culture of neural cells and many other cell types. Further, Matrigel has been used successfully when injected subcutaneously for studies of angiogenesis and revascularization [155]. The effects of Matrigel used as a biomaterial for brain tissue repair however, are still unclear. In animal models of stroke/injury, both Lu *et al.* [156] and Jin *et al.* [23] found that Matrigel reduced the volume of the stroke/injury lesion only when it encapsulated cells. Matrigel without cells was no better at reducing the lesion volume than cells alone or buffer-injected control brains. Unfortunately, neither of these studies evaluated the glial response to the Matrigel. A study by Uemura, *et al.* [20] however, demonstrated increased apoptosis of neural precursor cells encapsulated in Matrigel compared with unencapsulated control cells. In these studies, neural precursor cells encapsulated into growth-factor reduced Matrigel were implanted into the normal mouse striatum. The number of TUNEL positive cells was significantly higher in encapsulated cells than in unencapsulated cells for at least 72 h post-implant [20]. The preservation of the implanted cells is a primary function for biomaterials in brain tissue engineering. In addition, photomicrographs from reveal a dense core of peripheral leukocytes in the Matrigel with encapsulated cells [20]. This leukocyte reaction appears reduced in the cells alone group [20]. As with other studies, the authors did not evaluate biocompatibility with regards to the glial reaction. While these examples are not as dramatic as those recorded for poly(methylidene malonate 2.1.2) microspheres [1,2], it continues to underscore the need to ensure that the hydrogel and its components are designed to promote success both to the individual cells and to the surrounding tissues.

## 4. Physical Formation

Even if the basic chemistry is non-immunogenic, the physical characteristics of a hydrogel can contribute to the biocompatibility. Such characteristics as mesh size, presence or absence of pores, surface texture, and overall shape will have significant impact on how the hydrogel is accepted by the tissues of the CNS.

Whether the hydrogel is acting as a culture environment or implanted into neural tissue, fluid flow through the hydrogel and the exchange of nutrients are necessary for biocompatibility. These functions are dependent on the mesh size of the hydrogel [157]. The hydrogel mesh size, or distance between polymer crosslinks, must be sufficiently large enough for the free diffusion of gases and fluids and the movement of small molecules, such as growth factors. This allows the opportunity for encapsulated cells to receive nutrients and to rid the environment of wastes. Mesh size also contributes to the rate of degradation, with larger mesh sizes factoring in faster degradation [103,105]. A review by Kloxin *et al.* [31] provides a good overview of how the mesh size and cross-linking density of polymer structures impacts cell survival and growth.

Several methods of hydrogel fabrication also allow for the formation of pores within a hydrogel [158,159,160,161,162]. Pores can serve many purposes to enhance the functionality of a hydrogel. For example, pores can be incorporated to improve neurite extension or promote vascularization [159,160,161,162,163,164,165]. Namba *et al.* [162] demonstrated an increased number of neurites and neuritic branching from primary neurons encapsulated in PEG scaffolds with approximately 1.6 µm diameter pores compared with non-porous PEG scaffolds. While the presence of pores will likely contribute to the function and success of a hydrogel, there is little evidence that pores contribute directly to biocompatibility of encapsulated cells in small hydrogel scaffolds [160]. However, if a large volume of hydrogel is introduced into the brain, the existence of pores may allow for vascularization of the implanted hydrogel. To use vascularization of tumors as an example, an implant volume of greater than 1 to 2 mm^3^ would require new vascularization for encapsulated cell survival [166].

The use of microspheres, or microparticles, is a way to produce targeted drug delivery, both spatially and temporally. Microspheres can be designed to degrade in a manner or time scale which differs from the surrounding hydrogel. For instance, Lampe *et al.* [75], demonstrated the release of growth factors into the brain from poly(lactic-*co*-glycolic acid) (PLGA) microspheres at different rates based on PLGA carboxylation. Carboxylated PLGA microspheres released growth factor more quickly, through 28 days post-implantation, while standard PLGA microspheres released growth factor for more than 56 days post-implantation [75]. Despite the functional relevance of microspheres, they also reveal the impact of size and surface area on biocompatibility. Increased surface area generally means an increased immune response [60]. Thus, it is important to consider the volume and shape of the material is being implanted into the CNS tissue.

In designing a hydrogel to enhance or replace neural tissue, hydrogel engineers must keep in mind the desired site of implant and the function and specific anatomy of the surrounding tissue. These features will likely constrain the design choices for the hydrogel. For example, studies by Davies *et al.* [73] have shown that when endogenous fibrous components in the glia scar are aligned correctly, axonal outgrowth can be observed across the damaged spinal cord. The neuroarchitecture of the spinal cord is highly organized with parallel tracts of neurites and axons and thus, its repair may benefit from a hydrogel with organized architecture, such as nanofibers or nanotubes [33,167,168,169,170,171]. Implantation of hydrogels into white matter, such as in the spinal cord or corpus callosum, will limit access to free water and thus slow the hydrolytic degradation compared to grey matter [105,111,112,115]. Comparatively, a cerebral stroke cavity in the brain may require a hydrogel that can be injected prior to polymerizing but which polymerizes *in situ* to fill the amorphous cavity [5,23,25,27,101,172]. 

Surface texture, or topography, of a hydrogel material can also affect neural cell morphology and migration [170,173,174,175]. Particularly, neural stem cells undergo migration, development, and polarization patterns based on contact guidance cues, which can be mimicked though complex structuring of the polymer surface [170,174,175,176,177,178]. A grooved pattern can be used to guide neurite extension, however optimal neuritic growth is dependent on the depth and width of the groove [175]. Altering the texture of the surface also alters the total exposed area which is likely to lead to an increased immune response.

## 5. Mechanical Properties and Characteristics

Mechanical properties of biomaterials have been shown to play a greater role in the growth and development of CNS cells than previously realized. Neural cell processes, from attachment and migration to differentiation and maturation, have been shown to be dependent on culture substrate stiffness and elasticity [27,173,179,180,181,182]. Neural cells have been shown to grow best in softer hydrogels where the mechanical properties more closely match those found in native neural tissue [27,34,142,173,180,181,182,183,184,185,186]. Furthermore, the fates of these cells have also been shown to be dependent on hydrogel stiffness: Neurons have been shown to survive best on hydrogels with a stiffness less than 1kPa, while astrocytes survive better on relatively more stiff hydrogels, up to 10 kPa [27,34,94,180,181,182,184,185,186]. Designing a hydrogel for the replacement of dopamine neurons for Parkinson’s disease may require the less stiff mechanical properties necessary for neuronal survival and neurite extension. In comparison, a hydrogel designed for rebuilding a damaged spinal cord tract could be relatively stiffer to support astrocyte and oligodendrocyte survival [27,34,180,181,182,185,186,187]. Such subtle variations in mechanical properties impact the functionality of a hydrogel to guide encapsulated cell fate and survival, while larger variations can alter the biocompatibility of the material to brain or spinal cord as a whole.

Matching of the hydrogel mechanical properties to host tissue is important to minimize contact stresses and the possibility of an exacerbated immune response. The effects of differing mechanical properties between a tissue and implant are much better understood in peripheral tissues but have rarely been studied in soft tissues, such as the brain and spinal cord [188]. Therefore, it is relatively unknown what impact mechanical properties may have on CNS tissues. Biran *et al.* [122] demonstrated the effects of the incompatibility of chronically implanted silicon microelectrodes. A significant microglial response and loss of neuronal cells bodies in the region around the microelectrode illustrates a foreign body immune response in the brain. While the authors did not elucidate as to the exact cause of the neuroimmune response, it could easily be suggested that the difference in mechanical nature between the implant and surrounding tissue is likely to contribute to the immunorejection [122]. As stated previously, *in vitro* studies suggest that differing mechanical properties impact the survival of different neural cell types, with relatively stiffer hydrogels supporting astrocytes preferential to neurons [27,34,94,180,181,182,184,185,186]. This effect might also be observed *in vivo*, where, with a relatively stiffer hydrogel, a preference towards the support of astrocytes may contribute to a greater immune response. To promote biocompatibility and successful integration of a hydrogel into neural tissue, it might be best to mimic the mechanical properties of the brain (Table 1) and spinal cord (Table 2) and keep in mind the forces to which tissues of the CNS might be exposed (Table 3). For example, the spinal cord must be flexible enough to move, bend, or twist within the vertebrae under normal movement. However, under extreme conditions the spinal cord is susceptible to stretching, shearing, and compacting, as would be experienced during a car accident. 

Despite what we perceive is the importance of matching material mechanical properties, very few standards have been well developed for designing hydrogels to match soft tissues, especially tissues of the CNS. A review of ASTM standards and the data available in the literature suggests there is much variability and inconsistency in the testing strategies for characterizing hydrogels and neural tissue. While a discussion of these standards and results is beyond the scope of this review, Cheng *et al.* [189] and Chatelin *et al.* [190] have both extensively reviewed results of *in vitro* and *in vivo* characterization of the mechanical properties of neural tissue. This lack of standardization is perhaps best realized by the wide variety of methods available to characterize both polymer hydrogels and neural tissue samples. Table 1 and Table 2 illustrate a number of these methods, properties each method measures, relevant ASTM standards, and any applicable data acquired from neural tissue.

**Table 1:** Test methods and relative brain measures.

Test Method	Measured Property	ASTM Standards	Measured CNS data	Refs.
Magnetic Resonance Elastography	Shear Modulus	N/A	HUMAN: Grey 5.22 kPa;	[191]
			White 13.6 kPa @ 100 Hz	
	Shear Storage/ Loss Modulus		HUMAN: G' (Storage) = 1.13 kPa;	[192]
			G" (Loss) = 0.935 kPa @100 Hz	
	Shear Modulus		HUMAN: 3.5 kPa @ 83.33 Hz	[193,194]
			G'(Storage) Grey matter = 3.1 kPa	
			G'(Storage) White matter = 2.7 kPa @200 Hz	
Dynamic Testing	Shear Storage/ Loss Modulus	D4065	PRIMATE: G' = 0.6-1.1 kPa;	[194]
			G" = 0.35-0.6 kPa @ 9-10 Hz	
	Dynamic Elastic Modulus		HUMAN : E' = 67 kPa; E" = 26 kPa;	[195]
			E*=72 kPa @ 34 Hz	
			PRIMATE : E' = 91 kPa; E" = 54 kPa;	
			E* = 105 kPa @ 31 Hz	
	Dynamic Storage/ Loss Modulus		HUMAN: Storage: 0.39 kPa, 0.47 kPa, 0.65 kPa	[196]
			Loss: 0.075 kPa, 0.094 kPa, 0.190 kPa @ 0.1,	
			1 and 10 Hz respectively	
Compression Testing	Elastic Modulus	D1621, D695, D575	PORCINE 5.7 kPa @ 1 s^−1^;	[197]
			11.9 kPa @ 10 s^−1^; 23.8 kPa @ 50 s^−1^	
	Shear ModulusMooney Rivlin Material Model		BOVINE : 2.26 kPa	[198]
			(Equation constants for Mooney-Rivlin Model)	
	Elastic Modulus		PORCINE: 38.5 kPa	[199]
Tensile Testing	Elastic Modulus	D1708	PORCINE: 4.2 kPa @ .9 s^−1^;	[197]
			4.0 kPa @ 4.3 s^−1^; 18.6 kPa @ 25 s^−1^	
Pressure/Volume Testing	Elastic Modulus	N/A	PRIMATE (*in vivo*): 10.0-3.50 kpa	[200]
Relaxation Testing	Relaxation Modulus	D6048	HUMAN: 6.6 kPa @ 34 Hz	[196]
			PRIMATE: 10.3 kPa @ 31 Hz	
Indentation Testing	Shear Modulus	E2546	PORCINE: Grey Matter 0.74 kPa;	[201]*
			White Matter 1.03 kPa @ 0.1 mm/s	
	Elastic/Shear Modulus		RAT: 13-17 day old: 3.34 kPa;	[202]*
			43-90 day old: 1.72 kPa	
	Shear modulus and Relaxtion functions		PORCINE: Short Term:* In vivo* 1.88 kPa;	[203]
			*In Situ* 2.80 kPa; *In Vitro* 1.36 kPa	
			Long Term: *In vivo* 0.685 kPa;	
			*In Situ* 0.926 kPa; *In Vitro* 0.468 kPa	
Atomic Force Microscopy	Elastic/Shear Modulus	E42,	RAT (hippocampus): pyramidal layer:	[204]
			CA1 0.14 kPa; CA3 0.23 kPa	
		E2382	stratum radiatum: CA1 0.17 kPa;	
			CA3 0.31 kPa @ 3µm indentation	

* It should be noted that these authors calculated Shear Modulus from indentation data per a formula derived by Lee and Radok in 1960. This value is normally calculated using dynamic or oscillatory methods. ASTM standard provides guidelines for using indentation to calculate Elastic modulus.

**Table 2:** Test methods and relative spinal cord measures.

Test Method	Measured Property	ASTM Standards	Measured CNS data	Refs.
Tensile Testing	Elastic Modulus	D1621, D695, D575	HUMAN: With Pia Matter 1400 kPa @ 1 and 10 s^−1^;	[205]
			Without Pia Matter 89.0 kPa @ 1 and 10 s^−1^	
	Elastic Modulus		HUMAN: 1020 kPa @ 0.068 s^−1^;	[206]
			1170 kPa @ 0.14 s^−1^; 1370 kPa @ 0.21 s^−1^	
	Elastic Modulus		CANINE (juvenile,* in vivo*):	[207]
			265 kPa @ 0.021 mm/s	
	Elastic Modulus		FELINE (*In vivo*):	[208]
			400-2600 kpa @ 0.0003 s^−1^	
	Elastic Modulus		CANINE: 16.8-19.0 kPa, loaded discretely in	[209]
			5 g increments from 0 to 150 g	
	Elastic Tangent Modulus		BOVINE:	[210]
			White Matter: 1050 kPa @ 0.05 s^−1^, 40% strain	
			Grey Matter: 962 kPa @ 0.05 s^−1^, 40% strain	
Pressure/Volume Testing	Elastic Modulus	N/A	RABBIT:	[211]
			Axial Section: Grey 3.40 kPa; White 3.40 kPa	
			Frontal Section: Grey 3.00 kPa; White 0.003.50 kPa	
			Sagital Section: Grey 3.50 kPa; White 2.80 kPa	
Indentation Testing	Elastic Modulus	E2546	RAT: Healthy 0.128 kpa ; Injured: 0.69 kPa	[212]

**Table 3:** Material characteristics and scenarios of normal tissue exposure.

Material Characteristic	Loading Description	Possible Implications	Load Scenario
Creep/Relaxation Behavior	Constant Pressure	Permanent deformation	Static pressure on brain tissue from surrounding tissue, fluid, and bone
		Change in elastic modulus	
Fatigue Stress Behavior	Cyclic pressure	Permanent deformation	Normal intracranial pressure fluctuationsGlial cell migration through microenvironments
		Changes in material properties	
			(*i.e.*, white matter tracts/ spinal cord)
		Hardening/crystallization	
		Fracture/Failure	
Fatigue Strain Behavior	Cyclic deformation	Permanent deformation	Normal intracranial pressure fluctuations
		Changes in material properties	
		Hardening/crystallization	Motion in spinal cord within the vertebrae
		Fracture/Failure	
Stress/Strain Recovery	Tension or Compression	Permanent deformation	Flexion or extension in spinal cord within vertebrae
			Intracranial hyper/hypotension
			Normal intracranial pressure fluctuations

## 6. Discussion

Successful tissue engineering must first start with polymers which are biocompatible. Biocompatibility of polymer hydrogels is a complex interaction between the tissue in which it is implanted and all the properties of the hydrogel. While much work has been done establishing biocompatibility and testing standards for engineering tissues in the body, such as cartilage, very little has been done in the brain and spinal cord by comparison. Neural tissue engineering is further complicated by the fact that the brain and spinal cord have an immune response involving different triggers and predominately different cells than the peripheral immune system and by the limited ability of the CNS to self-regenerate. With this in mind, biocompatibility is an important issue in neural tissue engineering. Materials found to be biocompatible in the body are not, by default, biocompatible in the brain and spinal cord. This review sought to summarize the immune process in the CNS and then to discuss the polymer and hydrogel characteristics that might be most important for neural tissue engineering strategies.
